# IL-1α/IL-1R1 Expression in Chronic Obstructive Pulmonary Disease and Mechanistic Relevance to Smoke-Induced Neutrophilia in Mice

**DOI:** 10.1371/journal.pone.0028457

**Published:** 2011-12-06

**Authors:** Fernando M. Botelho, Carla M. T. Bauer, Donna Finch, Jake K. Nikota, Caleb C. J. Zavitz, Ashling Kelly, Kristen N. Lambert, Sian Piper, Martyn L. Foster, James J. P. Goldring, Jadwiga A. Wedzicha, Jennifer Bassett, Jonathan Bramson, Yoichiro Iwakura, Matthew Sleeman, Roland Kolbeck, Anthony J. Coyle, Alison A. Humbles, Martin R. Stämpfli

**Affiliations:** 1 Department of Pathology and Molecular Medicine, Centre for Gene Therapeutics, McMaster University, Hamilton, Canada; 2 Medical Sciences Graduate Program, McMaster University, Hamilton, Canada; 3 MedImmune LTD, Cambridge, United Kingdom; 4 Research and Development, AstraZeneca, Charnwood, United Kingdom; 5 Academic Unit of Respiratory Medicine, University College London, London, United Kingdom; 6 Center for Experimental Medicine and Systems Biology, Institute of Medical Science, University of Tokyo, Tokyo, Japan; 7 MedImmune LLC, Gaithersburg, Maryland, United States of America; 8 Department of Medicine, McMaster University, Hamilton, Canada; National Jewish Health, United States of America

## Abstract

**Background:**

Cigarette smoking is the main risk factor for the development of chronic obstructive pulmonary disease (COPD), a major cause of morbidity and mortality worldwide. Despite this, the cellular and molecular mechanisms that contribute to COPD pathogenesis are still poorly understood.

**Methodology and Principal Findings:**

The objective of this study was to assess IL-1 α and β expression in COPD patients and to investigate their respective roles in perpetuating cigarette smoke-induced inflammation. Functional studies were pursued in smoke-exposed mice using gene-deficient animals, as well as blocking antibodies for IL-1α and β. Here, we demonstrate an underappreciated role for IL-1α expression in COPD. While a strong correlation existed between IL-1α and β levels in patients during stable disease and periods of exacerbation, neutrophilic inflammation was shown to be IL-1α-dependent, and IL-1β- and caspase-1-independent in a murine model of cigarette smoke exposure. As IL-1α was predominantly expressed by hematopoietic cells in COPD patients and in mice exposed to cigarette smoke, studies pursued in bone marrow chimeric mice demonstrated that the crosstalk between IL-1α+ hematopoietic cells and the IL-1R1+ epithelial cells regulates smoke-induced inflammation. IL-1α/IL-1R1-dependent activation of the airway epithelium also led to exacerbated inflammatory responses in H1N1 influenza virus infected smoke-exposed mice, a previously reported model of COPD exacerbation.

**Conclusions and Significance:**

This study provides compelling evidence that IL-1α is central to the initiation of smoke-induced neutrophilic inflammation and suggests that IL-1α/IL-1R1 targeted therapies may be relevant for limiting inflammation and exacerbations in COPD.

## Introduction

The adverse effect of cigarette smoking on human health is well established [1], [2]. Cigarette smoking is a main risk factor for lung cancer and directly correlates with the development of chronic obstructive pulmonary disease (COPD). According to WHO estimates, 80 million people have moderate to severe COPD [3]. Episodes of acute exacerbation of COPD (AECOPD), often requiring hospitalization, are typically caused by viral and/or bacterial infections. While aberrant immune-inflammatory responses are likely key for driving increased inflammation observed in COPD patients during stable disease and episodes of acute exacerbations, the precise cellular and molecular mechanisms remain to be elucidated.

IL-1 is a key family of cytokines implicated in both initiation and persistence of inflammation [4]. Signaling through the IL-1 receptor type 1 (IL-1R1), IL-1α and IL-1β promote the production of pro-inflammatory cytokines. For maximal activity to be achieved, IL-1β requires processing by caspase-1 (within the inflammasome complex), caspase-8, or MMP-9 [5], [6], [7], while IL-1α is bioactive as either its pro- or processed form [4]. In man, expression of IL-1β, in particular, has been shown to be increased following cigarette smoke exposure [8] and in primary explant cultures of bronchial epithelial cells derived from COPD patients [9]. In animal models, we and others have shown that cigarette smoke exposure significantly up-regulates IL-1β [10], [11]. More recently, Doz and colleagues demonstrated that cigarette smoke-induced inflammation was dependent on IL-1R1 signaling [12], while studies using caspase inhibitors have proposed an involvement of the inflammasome and IL-1 cytokine maturation [11], [12]. To-date, no studies in humans or mice have examined expression and function of IL-1α in inflammatory processes associated with cigarette smoke exposure and inflammatory exacerbation of stable COPD, despite the multiple roles the IL-1 family members have been shown to play in a multitude of inflammatory conditions (reviewed in [13]).

Based on the aforementioned studies, we assessed expression of IL-1α in COPD patients and investigated its functional relevance to inflammatory processes elicited by cigarette smoke in mice. Here, we show that IL-1α is expressed in stable COPD, and increases in a correlative fashion with IL-1β during COPD exacerbations. In mice, we demonstrate that smoke-induced neutrophilic inflammation is dependent on IL-1α, but independent of IL-1β. Furthermore, we provide evidence that IL-1R1 expression on structural cells is necessary and sufficient to elicit smoke-induced neutrophilia. Finally, our data suggest that IL-1α/IL-1R1-dependent activation of the airway epithelium is key for driving the exacerbated inflammation following H1N1 influenza A infection of smoke-exposed animals.

## Results

### IL-1α is increased in COPD patients and correlates with increased IL-1β levels

**Figure 1:**
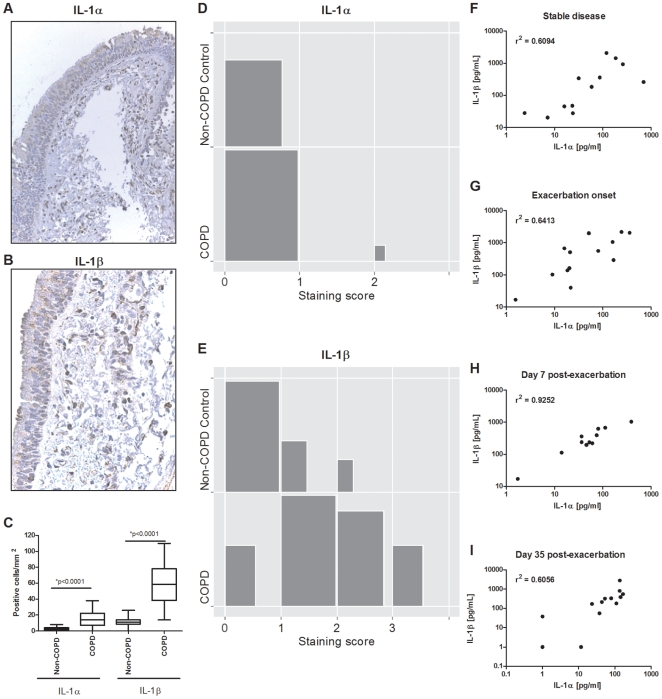
IL-1α and β are increased in the lung of chronic obstructive pulmonary disease patients. Representative images of IL-1α (A) and β (B) expression in lung biopsies from GOLD I/II COPD patients. (C) Positive cells were enumerated from two biopsy samples obtained from each patient (n = 5 non-COPD and n = 9 COPD GOLD stage I/II patients). Statistical significance was determined using a Generalized Linear Mixed Effect model with negative binomial (adjusted for dispersion) to take into account multiple sampling of the same patient. Whiskers of box plots represent 1–99 percentile. Lung sections from the same biopsy samples were scored for IL-1α (D) and β (E) staining in the epithelium as follows: 0, no staining; 1, occasional staining; 2, marked focal staining; 3, marked diffuse staining. A stratified Wilcoxon Ranksum test was used to compare the frequencies of the staining categories (0, 1, 2, and 3) and represented graphically (size of block is proportional to frequency). Levels of IL-1α and β were measured in sputum samples obtained from patients at enrolment during stable disease (F), at onset of exacerbation (G), and days 7 (H) and 35 (I) post-exacerbation.

Cigarette smoke elicits an inflammatory response in the lung which is a distinctive feature of COPD [14]; moreover, inflammation is further increased during episodes of AECOPD [15], [16]. The molecular mechanisms driving these inflammatory responses remain poorly understood. Since the IL-1 family of cytokines is important to the initiation of an inflammatory response, we investigated the expression of IL-1α and IL-1β in the lung of GOLD I/II COPD patients (see Table S1 for patient demographics in the data supplement). Lung section biopsies stained positively for both IL-1α and β (Figure 1A and B, respectively, and see Figure S1 in data supplement for control stains). Significantly increased numbers of IL-1α and β positive cells were enumerated in biopsy samples taken from GOLD I/II COPD patients compared to non-COPD controls (Figure 1C). IL-1α expression was confined largely to the inflammatory infiltrate, predominately on macrophages and the occasional granulocyte. By contrast, IL-1β expression was more widely distributed. Positive cells within the lamina propria were predominately macrophages with a few granulocytes. While IL-1α was not increased in the epithelium of COPD patients compared to non-COPD controls, IL-1β staining was significantly increased (p<0.0001) (Figure 1D and E, respectively). IL-1β positive cells included structural cells within the epithelial segment, notably metaplastic epithelial forms in both the airway epithelium and, occasionally, glandular epithelium.

Although it has been established that IL-1β levels are significantly increased in sputum recovered from COPD patients, no study, to our knowledge has examined IL-1α levels. In an exploratory study in COPD patients, levels of IL-1α and β recovered from the sputum of COPD patients were significantly correlated (p<0.0001) during stable disease, at the onset of exacerbation (prior to additional treatment), and 7 and 35 days post-exacerbation (Figure 1F–I). Correlation between IL-1α and β was strongest at 7 days post exacerbation. In a subset of patients, levels of IL-1α and β were increased at exacerbation compared to levels measured during the stable disease visit. Taken together, these data suggest a role for the IL-1 family members in stable disease and during episodes of acute exacerbation.

### IL-1α and IL-1β are increased in a model of cigarette smoke exposure

**Figure 2:**
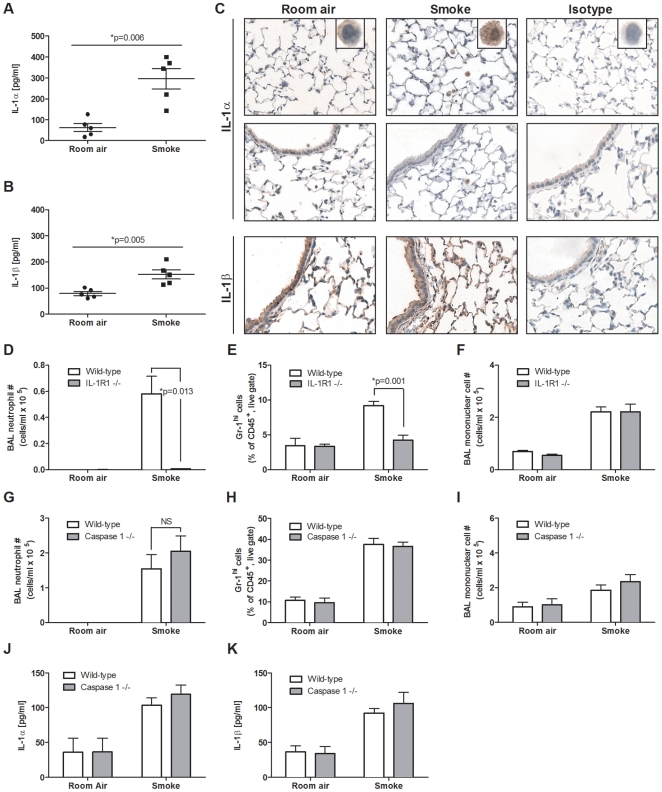
IL-1α and β expression in a smoke exposure model that induces IL-1R1-dependent and caspase-1-independent neutrophilia. BALB/c mice were room air or cigarette smoke-exposed for 4 days. Total levels of IL-1α (A) and β (B) protein were measured by ELISA in lung homogenates (n = 5 mice per group). (C) Representative images showing expression of IL-1α and β in room air and smoke-exposed BALB/c mice. Insets represent macrophages from the interstitial space. Wild-type and either IL-1R1-deficient (C57BL/6 background) (n = 5 mice per group) (D–F) or caspase-1-deficient (G–I) mice (NOD/ShiLt background) (n = 3–6 mice per group) were room air or cigarette smoke-exposed for 4 days. Neutrophils (D and G) and mononuclear cells (F and I) were assessed in the broncho-alveolar lavage (BAL) of room air and smoke-exposed mice. Percentage of Gr-1^hi^ cells were assessed by flow cytometric analysis of room air and smoke-exposed whole lung tissue from wild-type and either IL-1R1-deficient (E) (n = 5 mice per group) and caspase-1-deficient (H) (n = 4–6 mice per group) animals. Total levels of IL-1α (J) and β (K) protein were measured by ELISA from lung homogenates of room air and smoke-exposed wild-type and caspase-1-deficient mice (n = 4–6 mice per group). All data (B–K) are representative of two independent experiments.

To elucidate the functional importance of both IL-1α and IL-1β in inflammatory processes associated with smoking, we examined whether these cytokines were expressed in a mouse model of cigarette smoke exposure. BALB/c mice were exposed to room air or cigarette smoke twice daily for 4 days. Significantly increased levels of IL-1α and IL-1β were observed in the lungs of cigarette smoke-exposed mice compared to room air controls (Figure 2A and B).

Immunohistochemical analysis revealed that expression of IL-1α in control mice (room air) was confined to macrophages within the alveolar spaces (Figure 2C). Occasionally, a low grade staining was noted on intra-epithelial cells within the bronchiolar mucosa, bronchiolar epithelial cells, and epithelial secretory cells. In smoke-exposed mice, the key histological phenotype was a marked IL-1α expression on the expanded alveolar macrophage population (Figure 2C); although, IL-1α staining was also noted on some hyperplastic bronchiolar epithelial cells (data not shown). Of note, infiltrating cells within the bronchiolar and vascular adventitia compartments were IL-1α negative.

In contrast to the IL-1α expression pattern, widespread tissue expression of IL-1β was observed in room air and smoke-exposed mice (Figure 2C). In room air controls, variable expression was noted on the alveolar macrophage population. In addition, there was unequivocal expression on alveolar type (AT) I and ATII cells, especially the latter in the terminal alveolar buds, and on the occasional hypertrophic ATII cell. In smoke-exposed animals, a marked staining in the expanded alveolar macrophage population was observed, as well as increased expression in both the ATI and ATII cells, especially the hypertrophic forms. Widespread and marked expression of IL-1β was also observed on the bronchiolar epithelium, especially on hypertrophic cells, as well as epithelial secretory cells. Collectively, these data indicate that tissue expression of IL-1α and β in smoke-exposed mice involves a similar population of both inflammatory infiltrate and resident cells to that seen in COPD patients.

### Cigarette smoke-induced inflammation is driven independently of caspase-1

As the expression profile of IL-1α and β in cigarette smoke-exposed mice mirrored the clinical observations shown in Figure 1A and B, we used this experimental model as a platform to examine the functional importance of IL-1α and IL-1β to cigarette smoke-induced inflammation. We first exposed IL-1R1-deficient and C57BL/6 wild-type mice to cigarette smoke for 4 days to assess the reported dependence of IL-1R1 signaling to smoke-induced neutrophilic inflammation [11], [12]. Neutrophilia was completely attenuated in the bronchoalveolar lavage (BAL) and in the tissue of IL-1R1 deficient animals compared to wild-type controls (Figure 2D and E, respectively). An IL-1R1 deficiency did not impact the increase in mononuclear cells in the BAL of smoke-exposed mice (Figure 2F). The observed increase in mononuclear cells was the result of increased numbers of macrophages, as cigarette smoke exposure did not lead to an increase in BAL lymphocytes (data not shown). While the expression of neutrophil recruiting chemokines, CXCL -1, -2, and -5 were increased following smoke-exposure of wild-type mice, an IL-1R1 deficiency significantly attenuated their induction (Table S2 in online data supplement).

Given that caspase-1 cleaves pro-IL-1β into its bio-active form and that this process has been shown to contribute to cigarette smoke-induced neutrophilic inflammation [11], [12], we exposed caspase-1 deficient (NOD/ShiLt background) and the corresponding wild-type control mice to cigarette smoke for 4 days. Surprisingly, caspase-1 deficiency did not significantly alter smoke-induced neutrophilia in the BAL or tissue (Figure 2H and I, respectively). Similarly, the number of mononuclear cells in the BAL was not attenuated in smoke-exposed caspase-1 deficient mice compared to wild-type controls (Figure 2I). Of note, we observed similar levels of total IL-1α and IL-1β protein in wild-type and caspase-1 deficient mice (Figure 2J and K, respectively).

### Cigarette-smoke induced neutrophilia is IL-1α dependent

**Figure 3:**
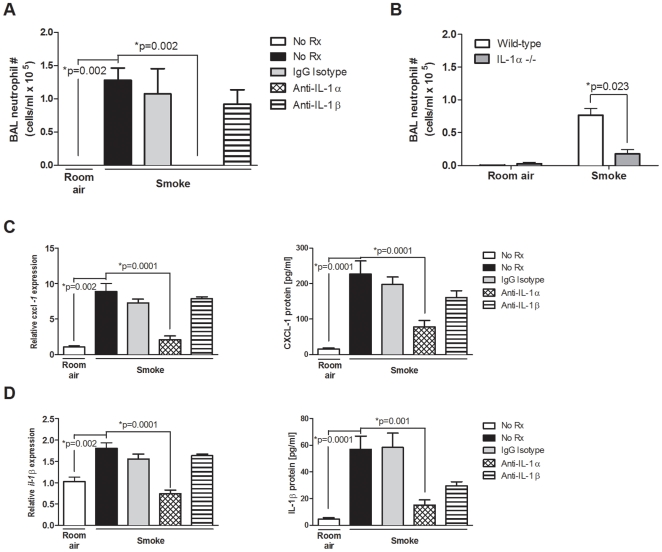
Cigarette smoke-induced neutrophilia is IL-1α dependent and IL-1β-independent. (A) 4 day cigarette smoke-exposed BALB/c mice were either left untreated (No Rx), or administered an isotype antibody (IgG isotype), or either an anti-IL-1α or anti-IL-1β blocking antibody. Neutrophil numbers were enumerated in the BAL (n = 4–5 mice per group from one of two independent experiments). (B) Wild-type and IL-1α-deficient mice were room air or cigarette smoke-exposed for 4 days. Data show BAL neutrophil numbers (n = 6–8 mice per group). CXCL-1 (C) and IL-1β (D) expression (left panels) were assessed in cigarette smoke-exposed and room air control BALB/c mice that were left untreated (No Rx), administered an isotype control antibody (IgG isotype), or either an anti-IL-1α or anti-IL-1β blocking antibody. Expression of *cxcl-1* and *IL-1β* transcripts was assessed by fluidigm array and is presented relative to no treatment room air control animals (n = 5 mice per group from one of two independent experiments). Total protein levels (right panels) of CXCL-1 (C) and IL-1β (D) were measured by MSD (n = 10 mice per group from two independent experiments).

IL-1R1 is the cognate receptor for both IL-1α and IL-1β [4]. Given the IL-1R1 dependency, we next investigated the relative importance of IL-1α and IL-1β to smoke-induced neutrophilic inflammation. We administered an anti-IL-1α or anti-IL-1β blocking antibody, or an isotype control antibody daily to cigarette smoke-exposed BALB/c mice. While anti-IL-1α intervention abrogated smoke-induced neutrophilia (Figure 3A), neither anti-IL-1β blockade nor administration of an isotype control impacted cigarette smoke-induced inflammation. To corroborate these findings, we exposed IL-1α-deficient and C57BL/6 wild-type control mice to cigarette smoke for 4 days. Significantly decreased numbers of neutrophils were observed in cigarette smoke-exposed IL-1α-deficient mice compared to wild-type controls (Figures 3 B), confirming our observations using blocking antibodies.

Since IL-1α significantly attenuated neutrophil recruitment to the lung of smoke-exposed mice, we assessed if neutrophil recruiting chemokines were preferentially attenuated by anti-IL-1α blockade. CXCL-1 had significantly increased RNA transcript and protein expression following cigarette smoke-exposure (Figure 3C). Anti-IL-1α, but not anti-IL-1β intervention significantly attenuated expression of CXCL-1 RNA transcripts and protein in smoke-exposed mice. Isotype antibody delivery did not impact transcript or protein expression levels. Furthermore, CXCL-2 and CXCL-5 gene expression, while increased following smoke-exposure, were attenuated by anti-IL-1α, but not anti-IL-1β blockade (Table S3 in the data supplement). Together, these data suggest that the neutrophilic inflammation observed in smoke-exposed animals requires the expression of CXCL -1, -2, and -5, which are attenuated by blockade of IL-1α, but not IL-1β.

As both IL-1α and IL-1β signal through the IL-1R1, we next studied whether IL-1α blockade attenuated expression of IL-1β. Figure 3D shows significantly decreased IL-1β transcript and protein levels in cigarette smoke-exposed mice that received anti-IL-1α antibody. Similarly, we observed decreased expression of GM-CSF, a cytokine that has recently been implicated in cigarette smoke-induced inflammation [17], [18] (refer to Table S3 in the data supplement). Finally, as also shown in Table S3 in the data supplement, we found that anti-IL-1α, but not anti-IL-1β blockade significantly attenuated expression levels of the macrophage elastase MMP-12. Collectively, these data suggest a critical role for IL-1α, but not IL-1β in mediating cigarette smoke-induced neutrophilia.

**Figure 4:**
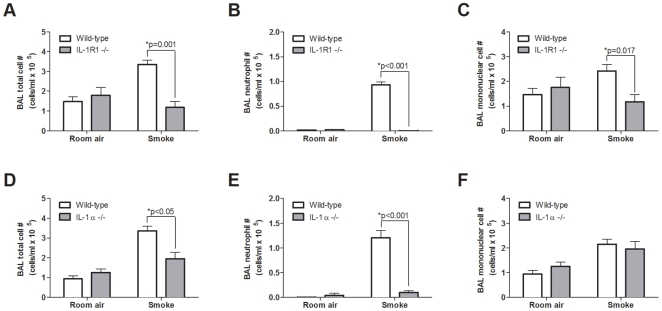
Neutrophilia induced by chronic cigarette smoke exposure is IL-1R1/IL-1α dependent. Wild-type C57BL/6 and either IL-1R1- (A–C) or IL-1α- (D–F) deficient mice were room air or cigarette smoke exposed for 8 weeks. Data show BAL total cell numbers (A and D), neutrophils (B and E), and mononuclear cells (D and F) (A–C: n = 4–5 mice per group from one of two independent experiments; D–F: n = 5 nice per group).

We next investigated whether neutrophilic inflammation observed following chronic cigarette smoke exposure (8 weeks) was also IL-1R1/IL-1α dependent. Decreased total cell numbers were observed in both IL-1R1- and IL-1α-deficient mice following chronic cigarette smoke exposure (Figures 4A and D, respectively). Similar to the acute exposure protocol, smoke-induced neutrophilia was significantly attenuated in both IL-1R1- and IL-1α-deficient animals compared to C57BL/6 wild-type controls (Figures 4C and F, respectively). Of note, we observed decreased mononuclear cell numbers in IL-1R1 deficient mice (Figure 4C), while no decrease was observed in IL-1α deficient animals. Collectively, these data provide clear evidence that cigarette smoke-induced neutrophilia is IL-1α dependent in both acute and chronic cigarette smoke exposure models.

### Expression of IL-1R1 on radio-resistant non-hematopoietic cells is required for smoke-induced inflammation

**Figure 5:**
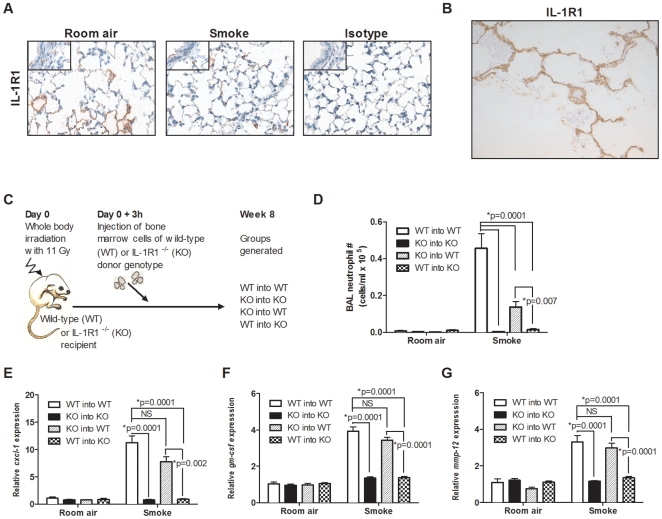
Expression pattern of IL-1R1 in smoke-exposed mice and COPD patients: requirement on radio-resistant non-hematopoietic cells. (A) IL-1R1 expression in representative images from room air and smoke-exposed (4 days) C57BL/6 mice. (B) Representative image of IL-1R1 expression as assessed in lung biopsies obtained from GOLD III COPD patients (see Figure S2 in the data supplement for isotype stains). (C) Various chimeric mice (coded as bone marrow donor genotype into recipient genotype) were generated. (D) Neutrophils were enumerated from the broncho-alveolar lavage (BAL) of bone marrow chimeric mice exposed to room air or cigarette smoke for 4 days (n = 5–7 mice per group). Expression of *cxcl-1* (E), *gm-csf* (F), and *mmp-12* (G) were measured by fluidigm array (n = 6–8 mice per group). All data are representative of one of two independent experiments.

As the IL-1R1 was shown to play a fundamental role in transducing the signals required for the initiation of acute and chronic smoke-induced neutrophilia, we sought to examine the expression profile of this receptor in the lung. Constitutive expression of the IL-1R1 was primarily observed within the alveolar epithelium of both ATI and ATII cell types (Figure 5A, inset) in room air and cigarette smoke-exposed mice. A similar expression pattern was mirrored in histological samples obtained from COPD patients (Figure 5B and refer to Figure S2 in the data supplement for control stains); although there was a clear expression on alveolar wall mesenchymal elements, which probably reflects enhanced remodeling in the clinical population as well as greater heterogeneity of lesion. Collectively, these data indicate that the alveolar epithelium is expressing IL-1R1 in both humans and mice, while hematopoietic cells within the alveolar space express IL-1α.

We thus hypothesized that crosstalk between hematopoietic and non-hematopoietic cells is essential for cigarette smoke-induced inflammation. To test this, we generated IL-1R1-deficient bone marrow chimeric mice. Bone marrow cells from C57BL/6 wild-type or IL-1R1-deficient mice were transferred intravenously to irradiated wild-type or IL-1R1-deficient recipient mice (Figure 5C). Following 8 weeks of reconstitution, mice were exposed to cigarette smoke for 4 days and inflammatory parameters assessed. Wild-type animals that received wild-type bone marrow cells (WT into WT) developed robust neutrophilia in response to cigarette smoke exposure (Figure 5D), whereas neutrophilia was absent in IL-1R1-deficient animals reconstituted with IL-1R1-deficient bone marrow cells (KO into KO). Chimeric mice, that resulted from the transfer of wild-type hematopoietic cells into irradiated IL-1R1-deficent mice (WT into KO), failed to elicit a neutrophilic response to smoke, suggesting that IL-1R1 expression on non-hematopoietic radio-resistant cells was essential for cigarette smoke-induced inflammation. Finally, transfer of IL-1R1-deficient hematopoietic cells into irradiated wild-type recipient mice (KO into WT) showed a significant, but partial reduction in cigarette smoke-induced neutrophilia.

We also investigated the expression of various genes, including, CXCL-1, GM-CSF, and MMP-12 (Figure 5 E–G, respectively), all of which were significantly attenuated in IL-1R1 deficient animals reconstituted with IL-1R1 deficient bone marrow cells (KO into KO). Interestingly, while cigarette smoke-exposed WT into KO chimeric animals had significantly attenuated gene expression, KO into WT animals did not significantly attenuate the genes measured compared to WT into WT control animals. Therefore, these data suggest that IL-1R1 mediated activation of non-hematopoietic cells is a prerequisite for cigarette smoke-induced inflammation, while IL-1R1 expression on hematopoietic cells is required for maximal neutrophil infiltration.

### IL-1R1 deficiency attenuates the differential response of the smoke-exposed lung to viral stimulus

**Figure 6:**
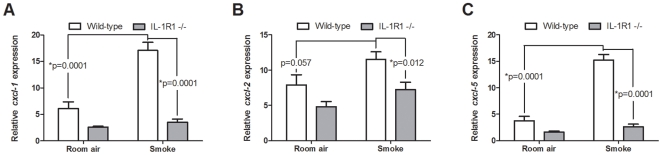
IL-1R1 deficiency in smoke-exposed precision cut lung slices attenuates lung resident responses to viral stimulus. Wild-type C57BL/6 and IL-1R1-deficient mice were exposed to room air or cigarette smoke for 4 days. PCLS were generated and stimulated *ex vivo* with a viral mimic, polyI:C. Expression of *cxcl-1* (A), *cxcl-2* (B), and *cxcl-5* (C) relative to room air control mock stimulated PCLS (data not shown) was assessed by real time quantitative RT-PCR (n = 7–14 lung slices from 3 independent experiments).

Having established that the IL-1α/IL-1R1 axis is central to smoke-induced inflammation, and that both IL-1α and β levels were increased in some patients during clinically unstable disease (refer to Figure 1F–I), we next assessed whether similar mechanisms may underlie the differential response of the smoke-exposed lung to viral challenge [19], [20], [21], [22]. We have previously reported using precision cut lung slices (PCLS) that the smoke-exposed lung is differentially primed to respond to viral insults [22]. Given the established importance of the epithelium in initiating smoke-induced neutrophilic inflammation, as demonstrated with the chimeric mouse experiments, we next sought to assess the impact of smoke exposure on the response of lung *resident* cells to viral stimulus. To this end, we exposed C57BL/6 wild-type and IL-1R1-deficient animals to cigarette smoke for 4 days and generated precision cut lung slices (PCLS). PCLS were stimulated *ex vivo* with the dsRNA ligand, polyinosinic polycytidylic acid (polyI:C), and expression of the key mediators were assessed. We observed a significantly greater induction in response to polyI:C stimulation of neutrophil recruiting chemokines, CXCL-1 and CXCL-5, and a modest increase in CXCL-2 from PCLS generated from smoke-exposed wild-type compared to room air-exposed controls (Figure 6). All transcripts measured were significantly attenuated in viral mimic-stimulated smoke-exposed IL-1R1-deficient PCLS. Collectively, these data demonstrate a role for lung *resident* cells in promoting smoke-induced inflammation and support a role for the IL-1R1 in the differential response of the smoke-exposed lung to viral infection.

### IL-1R1 deficiency and IL-1α antibody blockade attenuate inflammation during influenza infection of cigarette smoke-exposed mice

**Figure 7:**
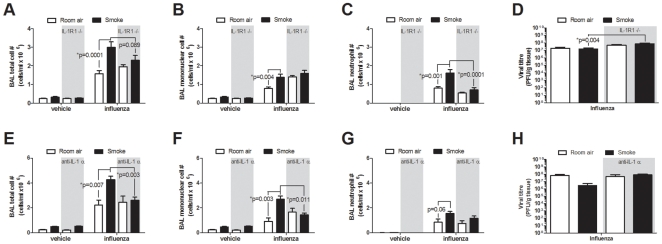
IL-1R1 deficiency and IL-1α antibody blockade attenuates inflammation in H1N1 influenza virus infected smoke-exposed mice. (A–D) Wild-type C57BL/6 or IL-1R1-deficient mice were exposed to room air or cigarette smoke for 4 days. Mice were then instilled with vehicle or infected with H1N1 influenza A virus. Five days post infection, total cell number (A), and mononuclear cell (B), and neutrophil (C) numbers were enumerated from the broncho-alveolar lavage (BAL) (n = 19–20 mice per group from four independent experiments) and viral burden was assessed (D) (n = 14–15 mice per group from three independent experiments). (E–H) Room air and smoke-exposed wild-type C57BL/6 mice treated daily with either isotype or IL-1α blocking antibodies were instilled with vehicle or infected with a H1N1 influenza A virus. Five days post-infection, total cell numbers (E), and mononuclear cell (F), and neutrophil (G) numbers were enumerated in the BAL and viral titers were assessed (H) (n = 4–5 mice per group).

Having established the importance of IL-1α in mediating signals via the IL-1R1 for the induction of smoke-induced inflammation, and given the role that *resident* cells of the smoke-exposed lung were shown to play in the response to viral mimic, we sought next to assess if these mechanisms underlie the exacerbated inflammatory response observed following viral infection *in vivo* [19], [20], [21], [22]. C57BL/6 wild-type and IL-1R1-deficient mice were exposed to cigarette smoke and subsequently infected with a H1N1 influenza virus. In agreement with previous observations [22], an exacerbated inflammatory response was observed in the BAL of cigarette smoke-exposed wild-type mice following viral infection compared to virally-infected room air control mice (Figure 7A). While IL-1R1 deficiency did not significantly affect total BAL inflammation in smoke-exposed influenza-infected mice (p = 0.089), neutrophilia was significantly attenuated in these animals compared to wild-type controls (Figure 7C). An IL-1R1-deficiency did not alter viral burden in room air-exposed, infected control mice, while more virus was recovered from the lungs of smoke-exposed IL-1R1-deficient mice compared to wild-type controls (Figure 7D). Taken together, these data suggest that an IL-1R1 dependent mechanism contributes to exacerbation of the inflammatory response in smoke-exposed mice following viral infection.

While an IL-1R1 deficiency could lessen exaggerated inflammatory responses in smoke-exposed influenza-infected animals, we hypothesized that IL-1α would play a predominate role in promoting this response. To test this, we injected animals daily with the anti-IL-1α or isotype antibodies during the course of cigarette smoke-exposure and viral infection. An exacerbated response to influenza A virus, in cigarette smoke-exposed C57BL/6 mice, was observed 5 days post-infection (Figure 7E). Anti-IL-1α neutralization markedly attenuated BAL total inflammation, with the effect significantly impacting mononuclear cells, but not neutrophils (Figure 7F and G, respectively). Interestingly, anti-IL-1α neutralization did not significantly impact viral burden in smoke-exposed or room air control mice (Figure 7H). Taken together these data provide evidence that therapies aimed at blocking IL-1α/IL-1R1 may be beneficial during periods of disease instability.

## Discussion

According to World Health Organization estimates, 80 million people have moderate to severe chronic obstructive pulmonary disease [3]. Although emphasis has been placed on reducing smoking prevalence, a greater understanding of the mechanisms that contribute to COPD pathogenesis and exacerbation of the stable disease are equally relevant given the burden this disease places on health care systems, and the addictive nature and chronic persistence of the cigarette smoking habit. Moreover, the efficacy of many existing anti-inflammatory interventions have been disappointing (reviewed in [23]), necessitating the design of novel therapeutics. Given that IL-1 family members play a central role in innate immune responses and in many pathogenic inflammatory disorders, we assessed in humans and mice the role of IL-1α and β, and their cognate receptor, IL-1R1, play in promoting cigarette smoked-induced inflammation.

In this study, we report the presence of both IL-1α and β in COPD patients. While previous studies have highlighted a role for IL-1β in disease progression [8], [9], we observed a strong correlation between IL-1α and IL-1β levels in sputum obtained from COPD patients in stable disease and during episodes of acute exacerbation. Mechanistically, we demonstrate using blocking antibodies and gene-deficient mice that cigarette smoke-induced neutrophilia, as well as, expression of the neutrophil recruiting chemokines CXCL -1, -2, and -5 was IL-1α dependent, but independent of IL-1β. IL-1α antibody intervention also attenuated expression of GM-CSF and MMP-12, two molecules that have been implicated in cigarette smoke-induced inflammation and tissue damage [17], [18], [24]. Our data therefore suggest a novel role for IL-1α in the pathogenesis of COPD.

Recently, macrophages have been proposed to respond to necrotic cells by releasing IL-1α [25]. Given that neutrophilia and pathological tissue damage, including necrosis are key hallmarks of COPD, it is plausible that IL-1α is released in COPD as a consequence of such mechanisms. We have previously shown that alveolar macrophages cultured *ex vivo* from smoke-exposed animals produce significantly more IL-1α compared to controls [26]. Thus, it would be of interest to examine the potential role of macrophages for contributing to IL-1α driven responses, especially given that macrophages have been suggested to be an orchestrating cell-type in COPD pathogenesis.

Our study suggests that caspase-1, the enzyme necessary for processing IL-1β into its active form [27], was dispensable for smoke-induced neutrophilic inflammation. Recently, two studies in mice showed that smoke-induced inflammation was dependent on signaling through the IL-1R1 [11], [12]. While, Doz and colleagues suggested that a caspase-1 inflammasome may be important to the activation of IL-1β, Churg *et al.* demonstrated using pharmacological caspase inhibitors that smoke-induced inflammation and increased serum levels of IL-1β could be attenuated. Although our observations are in contrast to those of Churg *et al.*, it remains to be addressed whether differences in mouse strain, period of smoke exposure, or the specificity of the pharmacological inhibitors are contributing factors. Interestingly, Chen *et al.* demonstrated *in vivo* that caspase-1 was redundant in the inflammatory response to dead cells [28], suggesting that this response is dependent on IL-1α but independent of IL-1β, and that necrotic events in cigarette smoke-exposed mice may be key for driving smoke-induced neutrophilic inflammation.

Interestingly, IL-1α blockade markedly attenuated IL-1β protein levels in the lung. Although not fully understood, this is an important observation as over-expression of IL-1β has been shown to contribute to emphysematous changes in mice [29]. Furthermore, a study by Castro *et al.* demonstrated that smoke-induced mononuclear cell infiltrates were attenuated with an anti-IL-1β antibody therapy [30]. Although IL-1β, in our model, does not appear to be necessary for cigarette smoke-induced inflammation, IL-1α up-regulation of IL-1β may provide a viable hypothesis for the difference in interventions and may potentially support the long term implications for the development of lung tissue damage in chronic pulmonary disease.

Given that the IL-1R1 was predominantly expressed on the airway epithelium and IL-1α was mainly restricted to hematopoietic cells, we addressed whether crosstalk between the hematopoietic and non-hematopoietic compartments was required for cigarette smoke-induced inflammation. Therefore, we generated IL-1R1-deficient bone marrow chimeric mice and showed that IL-1R1 signaling on non-hematopoietic structural cells, namely the alveolar epithelium, was essential for cigarette smoke-induced neutrophilic inflammation (Figure S3A in the data supplement), while IL-1R1 signaling on hematopoietic cells (presumably macrophages and dendritic cells) was required for maximal cellular inflammation (Figure S3B in the data supplement). Taken together, these data show that crosstalk, as depicted in Figure S3C in the online data supplement, between IL-1α^+^ hematopoietic cells (such as macrophages) and IL-1R1^+^ lung structural cells is key for driving the inflammatory response to cigarette smoke. Although IL-1α has been shown to drive inflammation via autocrine stimulation of cells [31], expression of IL-1R1 on hematopoietic cells (such as macrophages) is only partially involved in driving smoke-induced inflammation, suggesting that activation of non-hematopoietic cells by IL-1 is critical to the infiltration of neutrophils to the lung.

We and others have previously shown that cigarette smoke exposure exacerbates the inflammatory response elicited by influenza A viruses in mice [19], [20], [21], [22]. Given that lung epithelial cells are the primary target of respiratory viruses, we sought to assess whether IL-1R1 dependent activation of lung resident cells was mediating the the exacerbated inflammatory response. Indeed, IL-1R1 deficiency attenuated exacerbated neutrophilic responses in cigarette smoke-exposed influenza-infected animals. Using precision cut lung slices (PCLS) we provide clear evidence that the resident cells within the smoke-exposed lung produce greater levels of neutrophil recruiting chemokines, CXCL -1, -2, and -5 in response to a dsRNA stimulus. Of note, IL-1α blocking antibodies attenuated the exacerbated inflammatory response in smoke-exposed influenza-infected mice, suggesting that IL-1α blockade, while arguably having the potential to interfere with the host response to viral infection, in fact dampened the excessive inflammation observed in these animals. In line with observations reported by Schmitz and colleagues [32], we did not detect differences in viral burden between room air wild-type and IL-1R1-deficient mice. Interestingly, while no differences were observed in viral burden in anti-IL-1α antibody treated smoke-exposed mice, an IL-1R1-deficiency increased viral burden compared to wild-type controls. While it is well understood that an IL-1R1-deficiency increases influenza-related mortality at time-points later than were assessed in this paper [32], studies are on-going to examine the outcome of IL-1α blockade to antiviral responses (later than 5 days post-infection) in smoke-exposed mice.

In COPD, chronic infiltration of the lung by neutrophils is thought to play a key part in progression of the lung obstruction due to collateral tissue damage induced by these cells. During an exacerbation, release of further pro-inflammatory mediators and an over-exuberant neutrophilic response might contribute to progression of tissue damage and increase remodeling within the lung. Therefore, attenuation of pro-inflammatory responses to viruses may under these circumstances be beneficial. Collectively, these data provide evidence that blockade of IL-1α has a potential role in limiting disease exacerbation.

The results of our study demonstrate the importance of IL-1α and its cognate receptor, IL-1R1, to the induction of cigarette smoke-induced airway inflammation, and to the exacerbation of inflammatory processes following viral infection of smoke-exposed animals. Further, we identify that this response is independent of caspase-1 and thus redundant of IL-1β signaling of its receptor. Notably, crosstalk between IL-1α^+^ hematopoietic cells and non-bone-marrow derived IL-1R1^+^ cells, was found to be essential for governing smoke-induced inflammation. Collectively, these data support a role for IL-1α/IL-1R1 therapy in the management of smoke-induced inflammation and processes driving exacerbations of COPD.

## Materials and Methods

### Ethics Statement

All clinical samples were collected according to the principals of the Declaration of Helsinki, with patient informed written consent and local institutional ethical approval from the North West Research Ethics Committee, UK (application number 07/MRE08/42) and the Royal Free Hospital Ethics Committee, UK (reference number 06/Q0501/161). All human samples used in this study were managed via AstraZeneca R&D Charnwood Biobank facilities, and were analyzed anonymously by the authors. All animal experimentations were conducted in compliance with the Canadian Council on Animal Care and approved by The Animal Research Ethics Board at McMaster University (animal utilization protocol number 07-09-57).

### Human biopsies and sputum samples

Lung sections were obtained from biopsies taken from GOLD I and GOLD II COPD patients. Biopsy data from GOLD I (*n* = 3, 1 male and 2 females; all current smokers; mean ± SD of FEV_1_/FVC = 60±8%) and GOLD II (*n* = 6, 4 males and 2 females; 2 current smokers; mean ± SD of FEV_1_/FVC = 56±10%) COPD patients were combined. Normal samples of central airway (for comparison with COPD biopsy material) were all from non-smokers with no significant respiratory or co-morbidities.

Alveolar bed expression profiles (for IL-1R1) were assessed in lung sections obtained from GOLD III (*n* = 5, 3 males and 2 females; 1 current smoker; mean ± SD of FEV_1_/FVC = 44±10%) COPD patients. Data were compared with non-COPD materials obtained from cancer lobectomy from anatomically normal lobe regions. Lobectomies were all from former smokers who had ceased smoking for at least one year.

Sputum samples were obtained from COPD patients (demographics described in detail in Table S1 in the data supplement) at enrolment during stable disease, at onset of exacerbation, and 7 days and 35 days post-exacerbation. Exacerbation was defined as increase in two major symptoms (dyspnoea, sputum volume, or sputum purulence) or one major and one minor symptom (cough, wheeze, sore throat, nasal discharge, fever) over a 48 hr period. Patients were given a normal standard of care under the presenting circumstances, and sputum samples were taken at the discretion of the study investigator.

### Animals

BALB/c mice (6–8 weeks old) were purchased from Charles River Laboratories (Montreal, Canada). 6–8 weeks old IL-1R1-deficient mice (C57BL/6 background) and caspase-1-deficient mice (NOD/ShiLt background), as well as their respective wild-type controls were obtained from The Jackson Laboratories (Bar Harbor, ME, USA). IL-1α- deficient mice [33] (obtained from YI) were bred in-house. Mice were maintained under specific pathogen-free conditions in an access-restricted area, on a 12-h light-dark cycle, with food and water provided *ad libitum*.

### Cigarette smoke exposure

Mice were exposed to cigarette smoke twice daily, five days per week, using a whole body smoke exposure system (SIU-48, Promech Lab AB, Vintrie, Sweden) as previously described [10], [26]. Briefly, mice were exposed to 12 2R4F reference cigarettes with filters removed (Tobacco and Health Research Institute, University of Kentucky, Lexington, KY, USA) for a period of approximately 50 minutes, twice daily. Mice were exposed to cigarette smoke for either 4 days or 8 weeks as detailed in the results. This protocol of smoke exposure has been validated and shown to achieve blood carboxyhaemoglobin and cotinine levels that are comparable to those found in regular human smokers [10]. Control animals were exposed to room air only.

### Generation of IL-1R1-deficient bone marrow chimeric mice

5 million C57BL/6 wild-type or IL-1R1-deficient bone marrow cells were injected intravenously into irradiated (2 doses of 550Rads (11Gray total)) recipient C57BL/6 wild type (WT) or IL-1R1-deficient (knockout (KO)) mice. Recipient mice were on trimethoprim and sulfamethoxazole antibiotic-treated water one week prior to irradiation and two weeks following irradiation. Mice were allowed 8 weeks for reconstitution of hematopoietic bone marrow cells.

### Administration of antibodies and antibody inhibition studies

Mice were injected intraperitoneally with 400 µg of anti-IL-1α (clone ALF161; R&D Systems, Burlington, Canada), anti-IL-1β (clone B122; R&D Systems), or Armenian hamster isotype control antibody (Jackson Immunoresearch, Burlington, Canada) 12 hours prior to the first smoke exposure, and then daily 1 hour following the second smoke exposure. Influenza infected animals also received daily intraperitoneal injections during the course of infection.

Antibody inhibition studies (refer to Figure S4 in the data supplement) were carried out in bEnd3 cells (mouse endothelial IL-1 responsive cell line) that were cultured in DMEM, containing 10% FBS, 1% NEAA, sodium pyruvate, and 5 µM β-mercaptoethanol as per the supplier's recommendations. Cells were plated in 96 well plates and incubated at 37°C overnight. IL-1α or β (74 pM final assay concentration) was pre-incubated with titrations of antibodies at room temperature for 1 hr (2× final assay concentration) and then 100 µl was added to the cells, and incubated for 24 hrs at 37°C. Cell supernatants were diluted 1∶5 in PBS before being analyzed for murine KC using a standard KC ELISA as per the manufacturer's instructions (DY453, R&D Systems).

### Influenza infection and viral titer determination

Anesthetized mice were intranasally infected with 50 PFU of a mouse-adapted H1N1 influenza A (A/FM/1/47-MA) virus as previously described [22]. Control animals received 35 µl of PBS vehicle. A/FM/1/47-MA is a fully sequenced, plaque-purified preparation that is biologically characterized with respect to mouse lung infections [34]. Animals were not exposed to cigarette smoke on the day of viral delivery or for the entire course of the viral infection.

Lung homogenates were used to determine viral titre on Madin-Darby Canine Kidney (MDCK) monolayers. MDCK cells were maintained in α-MEM supplemented with 10% fetal bovine serum, 1% L-glutamine, and 1% penicillin/streptomycin. Confluent monolayers of MDCK cells were washed twice with PBS to remove serum. Serial dilutions of lung homogenate samples were prepared and 200 µl of each was added to the MDCK monolayers in 6 well dishes. Dishes were rocked and incubated at 37°C for 30 minutes. Agarose overlays (1∶1 ratio of 1.3% agarose (Biotechnology grade; Bioshop Canada INC., Burlington, Canada), and 2xMEM/F-11 supplemented with 2% L-glutamine and 2% penicillin/streptomycin, containing 1 µg/ml trypsin-EDTA) were applied and agarose allowed to solidify before replacing dishes to 37°C. Plaque assays were fixed using Carnoy's Fixative (75% methanol and 25% glacial acetic acid) two days later. Plaques were enumerated and viral titres were expressed as the number of infectious plaque forming units per gram of lung tissue.

### Collection and measurement of specimens

Bronchoalveolar lavage (BAL) fluid was collected, total cells enumerated, and differential cell counts obtained as previously described [10], [22], [26], [35].

### Flow cytometric analysis

Two lobes from the multilobe side of the lung was cut into ∼2 mm pieces, shaken for 1 h at 37°C in 150 U/ml collagenase type I (Gibco, Burlington, Canada) in HBSS, pressed through nylon mesh, washed twice in HBSS, and counted on a hemacytometer. Cells were washed, and re-suspended in 25 µl of staining cocktail for 30 min at 4°C. Antibodies used in this study include anti-CD45 (Allophycocyanin (APC)-cy7-conjugated, 30-F11, BD Biosciences, San Jose, CA, USA), anti-MHC class II (I-A/I-E) (APC-conjugated, M5/144.15.2, eBiosciences, San Diego, CA, USA), and anti-Gr-1 (Pacific Orange-conjugated, RB6-8C5, Invitrogen, Burlington, Canada), as well as appropriate isotype control antibodies (BD, San Jose, CA, USA and eBiosciences, San Diego, CA, USA). Fluorescence minus one staining cocktails and isotype control antibodies (BD Biosciences and eBiosciences) were used to distinguish background staining from positive signals. Flow cytometric data was acquired using an LSRII flow cytometer (BD Biosciences, San Jose, CA, USA). Data were analyzed with FlowJo Software (Tree Star, Ashland, OR, USA).

### Histological analysis and immunohistochemistry

For human expression of IL-1α and β, and IL-1R1 antigen retrieval was performed by incubating sections in 0.2% trypsin/0.2% CaCl_2_ in distilled H_2_O at 37°C for 10 minutes. Endogenous peroxidase activity was blocked using 6% H_2_0_2_ for 10 mins. To block non-specific binding of the secondary antibody, slides were incubated with 20% normal rabbit or goat serum for 20 minutes. Excess serum was removed and slides were incubated with either IL-1α rabbit anti-human antibody (Abcam, 9614, 2.5 µg/ml), IL-1β rabbit anti-human antibody (Abcam, 2105, 10 µg/ml) or IL-1RI goat anti-human antibody (R&D Systems, Ab-269-NA, 10 µg/ml) or either rabbit or goat IgG negative control for 1 hour. Slides were incubated with biotinylated rabbit anti-goat secondary (1∶200) or swine anti-rabbit secondary (1∶200) antibody for 20 minutes. Two antigen detection protocols were employed on the human tissue sets: 1) Strep ABComplex/HRP (Dako) for 20 minutes at room temperature, 2×10 minutes buffer wash and DAB applied for 1 minute. 2) Strep ABComplex/AP (Dako) for 30 minutes at room temperature, 2×10 minutes buffer wash and Fuchsin Substrate-Chromagen System (Dako) for 5 minutes. Slides were counterstained with haematoxylin (Sigma). Positive cells were counted from two separate biopsy samples from each patient taken approximately 10 µm apart. A 250 mm^2^ graticule was aligned to the basement membrane and cells counted in the lamina propria in 3 adjacent regions. Identification of positive cells was based on cytological morphology, comparing the relevant antibody stained sections with parallel haematoxylin and eosin stained sections. Many of the macrophages showed distinctive phagolysosome inclusions and granulated cytoplasm, whereas the granulocytes showed a distinctive granulated cytoplasm and typical nuclear morphology. Lung sections from the same biopsy samples were scored for IL-1α and β staining in the epithelium as follows: 0, no staining; 1, occasional staining; 2, marked focal staining; 3, marked diffuse staining.

For mouse expression of IL-1R1, 5 µg/ml of goat anti-mouse IL-1R1 antibody (R&D Systems, Minneapolis, MN, USA) was incubated on the slides for 1 hour. For the IL-1α and IL-1β stain, prior to the primary antibody incubation Rodent M Block (Biocare Medical, Concord, CA, USA) was added to each slide for 30 minutes, and then washed away with a Tris-buffered saline with 0.05% Tween-20 (TBS-T). 10 µg/ml of goat anti-mouse IL-1α and IL-1β (R&D Systems, Minneapolis, MN, USA) were prepared in Ultra Antibody Diluent (Thermo Scientific, Rockford, IL, USA) and incubated with the slides for 1 hour. A secondary goat polymer horse-radish peroxidase was used according to the manufacturer's instructions (BioCare Medical; Concord, CA, USA).

### RNA extraction for fluidigm analysis

RNA was extracted from mouse lung using the Qiagen RNeasy Fibrous Tissue kit (Qiagen, Hilden, Germany). RNA was quantified and normalized, and RNA integrity was assessed by Agilent Bioanalyzer using the Agilent RNA 6000 Nano Kit (Agilent, Santa Clara, CA, USA). cDNA generation was carried out with the Super Script III kit from Life Technologies utilizing the manufacturer's protocol (Life Technologies, Carlsbard, CA, USA). Relative transcript expression was assessed using the Fluidigm Biomark Dynamic array loaded with probes for transcripts of interest as previously described [36].

### ELISA and meso scale discovery (MSD) analysis

ELISA kits for IL-1α and IL-1β were purchased from R&D Systems (Minneapolis, MN, USA). Multi-array platform cytokine detection of KC and IL-1β was done using the multi-array murine pro-inflammatory and Th1/Th2 cytokine panel detection systems developed by Meso Scale Discovery (MSD; Gaithersburg, MD, USA).

### Precision cut lung slicing and culture

Lungs were sliced using a modification to a standard protocol that has previously been described [22]. Briefly, lungs were inflated with approximately 1.4 ml of agarose (type VII-A low gelling temperature; Sigma Aldrich, St. Louis, MO, USA) that was warmed to 37°C and prepared to a concentration of 2% in Hank's buffered saline solution (HBSS), supplemented with N-2-hydroxyethlypiperazine-N′-2-ethanesulphonic acid (HEPES) (0.2 M, pH 7.4). Subsequently, 0.2 ml of air was injected into the lung in order to flush the agarose-HBSS solution out of the conducting airways. The agarose was allowed to gel by cooling the lung to 4°C for 15 minutes. The lung lobes were dissected away and a flat surface was cut on the lobe parallel and caudal to the main bronchus. The lung lobes were maintained in an ice-cold 1× HBSS solution prior to and during slicing. 120 µm thick slices were generated using a vibratome (Leica; model VT 1000S, Richmond Hill, Canada) at 4°C. Approximately 40 slices were isolated from each mouse lung.

Lung slices were subsequently transferred to and cultured in Dulbecco's Modified Eagles Medium (DMEM)/F12 (Gibco, Burlington, Canada) supplemented with 35 µg/ml L-Ascorbic Acid (Sigma-Aldrich, Oakville, Canada), 5 µg/ml Transferin (Gibco, Burlington, Canada), 2.85 µg/ml Insulin (Sigma-Aldrich, Oakville, Canada), and 3.25 ng/ml Selenium (atomic absorption standard solution; Sigma-Aldrich, Oakville, Canada). The solution was filter-sterilized using a 0.22 µm pore filter. The DMEM/F12 solution was further supplemented with 250 ng/ml Amphotericin B (Sigma-Aldrich, Oakville, Canada) and 1% penicillin/streptomycin. The medium was changed every 1 h for the first 3 h of culture in order to remove any remaining agarose and cell debris from the lung slice culture. Lung slices were stimulated the next day for 6 hours with 100 ug/ml of dsRNA mimetic polyinosinic-polycytidylic acid (GE Healthcare, Mississauga, Canada) that was reconstituted in phosphate buffered saline or were left untreated. Samples were collected in RNA later (Ambion, Austin, TX, USA) and preserved at −80°C until extraction of RNA.

### RNA extraction and real-time quantitative RT PCR for precision cut lung slices

Lung slices were collected as previously described [22]. RNA was extracted from the lung slices according to the RNEasy Kit (Qiagen, Mississauga, ON, Canada). Optional on-column DNase digestion was performed during RNA extraction using the RNEasy Kit (Qiagen, Mississauga, ON, Canada). RNA was quantified using the Agilent 2100 Bio-analyzer (Agilent Technologies, Mississauga, ON, Canada). The quantity and integrity of isolated RNA was determined using the Agilent 2100 Bioanalyzer (Agilent, Palo Alto, CA, USA). Subsequently, 100 ng of total RNA was reverse-transcribed using 100 U of Superscript II (Invitrogen, Burlington, Canada) in a total volume of 20 µL. Random hexamer primers were used to synthesize cDNA at 42°C for 50 min, followed by 15 min incubations at 70°C. Real-time quantitative RT-PCR was performed in triplicate, in a total volume of 25 µl, using a Universal PCR Master Mix (Applied Biosystems, Foster City, CA, USA). Primers for CXCL-1, CXCL-1, CXCL-2, CXCL-5, GAPDH, along with FAM-labeled probes were purchased from Applied Biosystems. PCR was performed using the ABI PRISM 7900HT Sequence Detection System using the Sequence Detector Software version 2.2 (Applied Biosystems, Foster City, CA, USA). Data were analyzed using the delta, delta Ct method. Briefly, gene expression was normalized to the housekeeping gene (GAPDH) and expressed as fold change over the control group (room air control, mock).

### Data and statistical analysis

Data were presented and expressed as mean ± SEM using Graphpad Prism Software version 5 (La Jolla, CA, USA). Statistical analysis was performed with SPSS statistical software, version 17.0 (Chicago, IL, USA). For all human samples, statistical analysis were performed using R, version 2.11.1 (Vienna, Austria). In mouse experiments, we assessed significance (p<0.05) using the SPSS Univariate General Linear Model, t-tests were subsequently performed for two-group comparisons or one-way ANOVA with a Dunnett post-hoc test for multiple group comparisons, unless otherwise stated.

## Supporting Information

Figure S1 **IL-1α and β are increased in the lung of COPD patients.** Representative images showing expression of IL-1α (A) and β (B) isotype control stains as assessed in lung biopsy sections obtained from GOLD III COPD patients.
(TIF)

Figure S2 **IL-1R1 expression in the lung of a COPD patient.** Representative image of IL-1R1 isotype control stained lung biopsy section obtained from a GOLD III COPD patient.
(TIF)

Figure S3 **Model of IL-1α crosstalk between the hematopoietic and non-hematopoietic compartments.** (A) Absence of the IL-1R1 on non-hematopoietic cells, as depicted on the airway epithelium completely attenuates smoke-induced inflammation. (B) Absence of the IL-1R1 on hematopoietic (macrophages and dendritic cells) leads to attenuated neutrophilic inflammation that is not entirely abrogated. (C) IL-1R1 is required on cells in both the hematopoietic and non- hematopoietic compartments for cross-talk to occur and maximal smoke-induced neutrophilic inflammation to result.
(TIF)

Figure S4 **Antibody inhibition of IL-1 induced KC release from bEnd3 cells.** Murine IL-1α and β induced KC release was measured from bEnd3 cells, a murine endothelial cell line. (A) Murine IL-1β (74 pM) induced KC release was inhibited by MAB4012, and not by MAB4001 (B) Murine IL-1α (74 pM) induced KC release was inhibited by MAB4001, but not by MAB4012. Data shown is a single experiment representative of *n* = 3.
(TIF)

Table S1 **Study population characteristics, (** ***n*** **= 14), for sputum cohort.**
(DOC)

Table S2 **Expression profile of various mediators in IL-1R1-deficient animals.**
(DOC)

Table S3 **Expression profile of various mediators following anti-IL-1 –α and –β intervention.**
(DOC)

## References

[1] Patel RR, Ryu JH, Vassallo R (2008). Cigarette smoking and diffuse lung disease.. Drugs.

[2] Kim V, Rogers TJ, Criner GJ (2008). New concepts in the pathobiology of chronic obstructive pulmonary disease.. Proceedings of the American Thoracic Society.

[3] Holmes J (2010). 10 Facts on the Tobacco Epidemic and Global Tobacco Control.

[4] Dinarello CA (2009). Immunological and inflammatory functions of the interleukin-1 family.. Annu Rev Immunol.

[5] Schroder K, Tschopp J (2010). The inflammasomes.. Cell.

[6] Maelfait J, Vercammen E, Janssens S, Schotte P, Haegman M (2008). Stimulation of Toll-like receptor 3 and 4 induces interleukin-1beta maturation by caspase-8.. J Exp Med.

[7] Schönbeck U, Mach F, Libby P (1998). Generation of biologically active IL-1 beta by matrix metalloproteinases: a novel caspase-1-independent pathway of IL-1 beta processing.. J Immunol.

[8] Kuschner WG, D'Alessandro A, Wong H, Blanc PD (1996). Dose-dependent cigarette smoking-related inflammatory responses in healthy adults.. Eur Respir J.

[9] Rusznak C, Mills PR, Devalia JL, Sapsford RJ, Davies RJ (2000). Effect of cigarette smoke on the permeability and IL-1beta and sICAM-1 release from cultured human bronchial epithelial cells of never-smokers, smokers, and patients with chronic obstructive pulmonary disease.. Am J Respir Cell Mol Biol.

[10] Botelho FM, Gaschler GJ, Kianpour S, Zavitz CC, Trimble NJ (2009). Innate Immune Processes are Sufficient for Driving Cigarette Smoke Induced Inflammation in Mice.. Am J Respir Cell Mol Biol.

[11] Churg A, Zhou S, Wang X, Wang R, Wright JL (2009). The role of interleukin-1beta in murine cigarette smoke-induced emphysema and small airway remodeling.. Am J Respir Cell Mol Biol.

[12] Doz E, Noulin N, Boichot E, Guenon I, Fick L (2008). Cigarette smoke-induced pulmonary inflammation is TLR4/MyD88 and IL-1R1/MyD88 signaling dependent.. J Immunol.

[13] Sims JE, Smith DE.  The IL-1 family: regulators of immunity.. Nat Rev Immunol.

[14] Pauwels RA, Buist AS, Calverley PM, Jenkins CR, Hurd SS (2001). Global strategy for the diagnosis, management, and prevention of chronic obstructive pulmonary disease. NHLBI/WHO Global Initiative for Chronic Obstructive Lung Disease (GOLD) Workshop summary.. Am J Respir Crit Care Med.

[15] Aaron SD, Angel JB, Lunau M, Wright K, Fex C (2001). Granulocyte Inflammatory Markers and Airway Infection during Acute Exacerbation of Chronic Obstructive Pulmonary Disease.. Am J Respir Crit Care Med.

[16] Papi A, Bellettato CM, Braccioni F, Romagnoli M, Casolari P (2006). Infections and airway inflammation in chronic obstructive pulmonary disease severe exacerbations.. Am J Respir Crit Care Med.

[17] Vlahos R, Bozinovski S, Chan SP, Ivanov S, Linden A Neutralizing granulocyte/macrophage colony-stimulating factor inhibits cigarette smoke-induced lung inflammation.. Am J Respir Crit Care Med.

[18] Botelho FM, Nikota JK, Bauer C, Davis NH, Cohen ES A mouse GM-CSF receptor antibody attenuates neutrophilia in mice exposed to cigarette smoke.. Eur Respir J.

[19] Robbins CS, Bauer CM, Vujicic N, Gaschler GJ, Lichty BD (2006). Cigarette Smoke Impacts Immune Inflammatory Responses to Influenza in Mice.. Am J Respir Crit Care Med.

[20] Gualano RC, Hansen MJ, Vlahos R, Jones JE, Park-Jones RA (2008). Cigarette smoke worsens lung inflammation and impairs resolution of influenza infection in mice.. Respir Res.

[21] Kang MJ, Lee CG, Lee JY, Dela Cruz CS, Chen ZJ (2008). Cigarette smoke selectively enhances viral PAMP- and virus-induced pulmonary innate immune and remodeling responses in mice.. J Clin Invest.

[22] Bauer CMT, Zavitz CCJ, Botelho FM, Lambert KN, Brown EG (2010). Treating Viral Exacerbations of Chronic Obstructive Pulmonary Disease: Insights from a Mouse Model of Cigarette Smoke and H1N1 Influenza Infection.. PLoS ONE.

[23] Falk JA, Minai OA, Mosenifar Z (2008). Inhaled and Systemic Corticosteroids in Chronic Obstructive Pulmonary Disease.. Proc Am Thorac Soc.

[24] Hautamaki RD, Kobayashi DK, Senior RM, Shapiro SD (1997). Requirement for macrophage elastase for cigarette smoke-induced emphysema in mice.. Science.

[25] Rock KL, Latz E, Ontiveros F, Kono H.  The sterile inflammatory response.. Annu Rev Immunol.

[26] Gaschler GJ, Skrtic M, Zavitz CC, Lindahl M, Onnervik PO (2009). Bacteria challenge in smoke-exposed mice exacerbates inflammation and skews the inflammatory profile.. Am J Respir Crit Care Med.

[27] Li P, Allen H, Banerjee S, Franklin S, Herzog L (1995). Mice deficient in IL-1 beta-converting enzyme are defective in production of mature IL-1 beta and resistant to endotoxic shock.. Cell.

[28] Chen CJ, Kono H, Golenbock D, Reed G, Akira S (2007). Identification of a key pathway required for the sterile inflammatory response triggered by dying cells.. Nat Med.

[29] Lappalainen U, Whitsett JA, Wert SE, Tichelaar JW, Bry K (2005). Interleukin-1beta causes pulmonary inflammation, emphysema, and airway remodeling in the adult murine lung.. Am J Respir Cell Mol Biol.

[30] Castro P, Legora-Machado A, Cardilo-Reis L, Valenca S, Porto LC (2004). Inhibition of interleukin-1beta reduces mouse lung inflammation induced by exposure to cigarette smoke.. Eur J Pharmacol.

[31] Schultz K, Murthy V, Tatro JB, Beasley D (2007). Endogenous interleukin-1 alpha promotes a proliferative and proinflammatory phenotype in human vascular smooth muscle cells.. Am J Physiol Heart Circ Physiol.

[32] Schmitz N, Kurrer M, Bachmann MF, Kopf M (2005). Interleukin-1 is responsible for acute lung immunopathology but increases survival of respiratory influenza virus infection.. J Virol.

[33] Horai R, Asano M, Sudo K, Kanuka H, Suzuki M (1998). Production of mice deficient in genes for interleukin (IL)-1alpha, IL-1beta, IL-1alpha/beta, and IL-1 receptor antagonist shows that IL-1beta is crucial in turpentine-induced fever development and glucocorticoid secretion.. J Exp Med.

[34] Brown EG (1990). Increased virulence of a mouse-adapted variant of influenza A/FM/1/47 virus is controlled by mutations in genome segments 4, 5, 7, and 8.. J Virol.

[35] Gaschler GJ, Zavitz CC, Bauer CM, Skrtic M, Lindahl M (2008). Cigarette smoke exposure attenuates cytokine production by mouse alveolar macrophages.. Am J Respir Cell Mol Biol.

[36] Yao Y, Higgs BW, Morehouse C, De Los Reyes M, Trigona W (2009). Development of Potential Pharmacodynamic and Diagnostic Markers for Anti-IFN-α Monoclonal Antibody Trials in Systemic Lupus Erythematosus.. Human Genomics and Proteomics.

