# Supplementary figures and images for: IL-1α/IL-1R1 Expression in Chronic Obstructive Pulmonary Disease and Mechanistic Relevance to Smoke-Induced Neutrophilia in Mice
Source: PLoS One. 2011 Dec 6;6(12):e28457. doi: 10.1371/journal.pone.0028457 (PMC3232226; doi:10.1371/journal.pone.0028457)

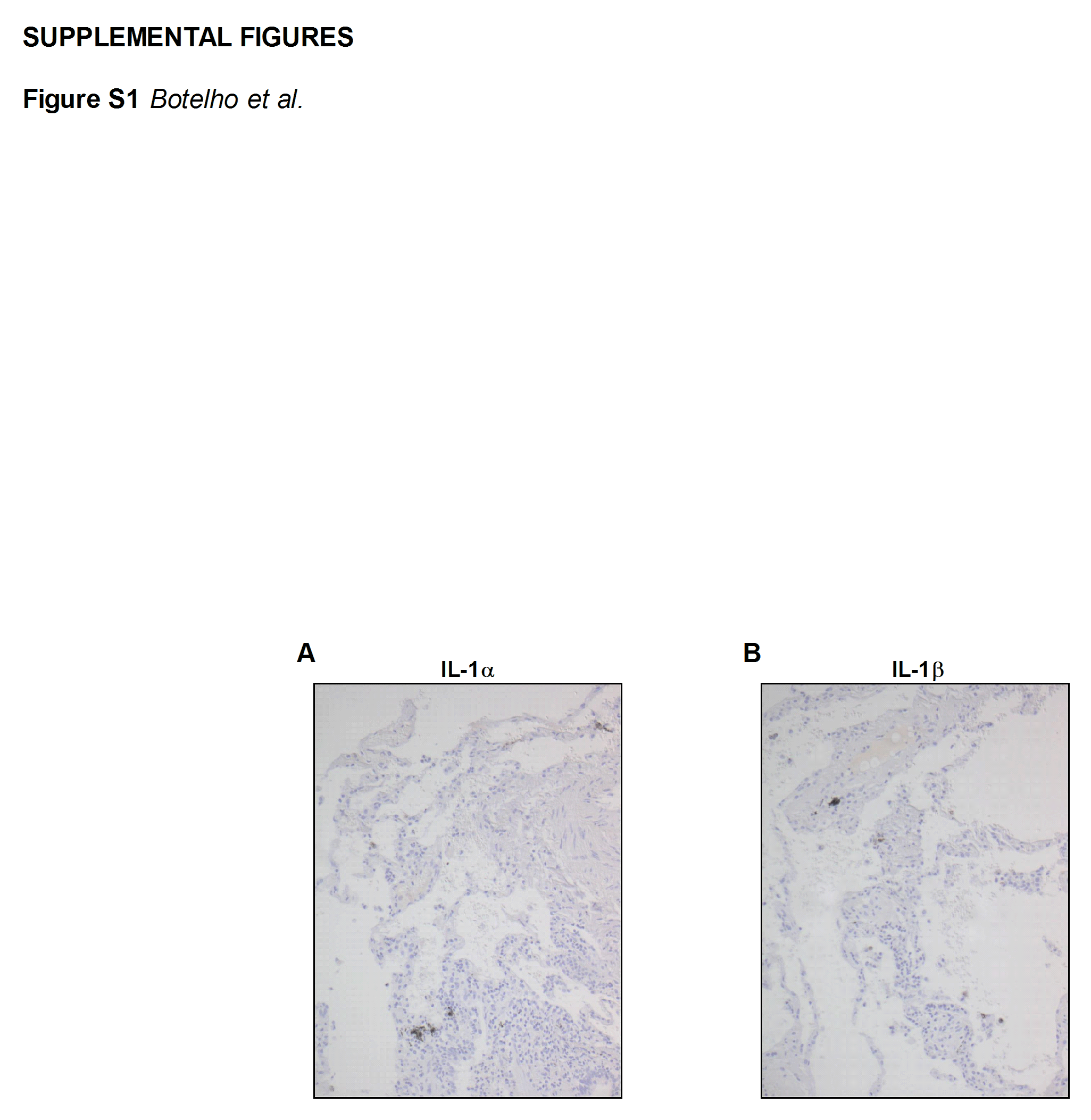

Supplement: Figure S1 — IL-1α and β are increased in the lung of COPD patients. Representative images showing expression of IL-1α (A) and β (B) isotype control stains as assessed in lung biopsy sections obtained from GOLD III COPD patients. (TIF) [file pone.0028457.s001.tif]

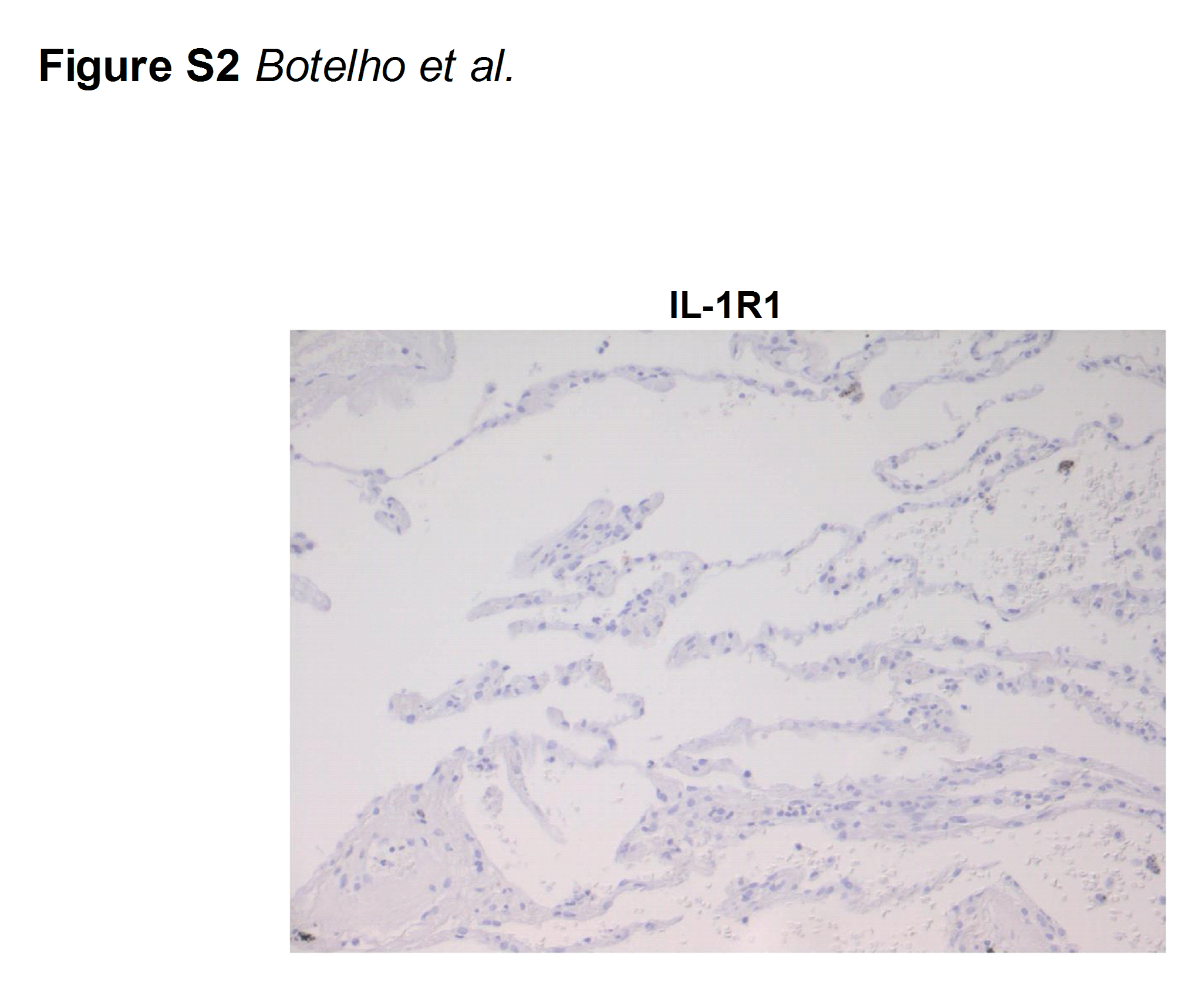

Supplement: Figure S2 — IL-1R1 expression in the lung of a COPD patient. Representative image of IL-1R1 isotype control stained lung biopsy section obtained from a GOLD III COPD patient. (TIF) [file pone.0028457.s002.tif]

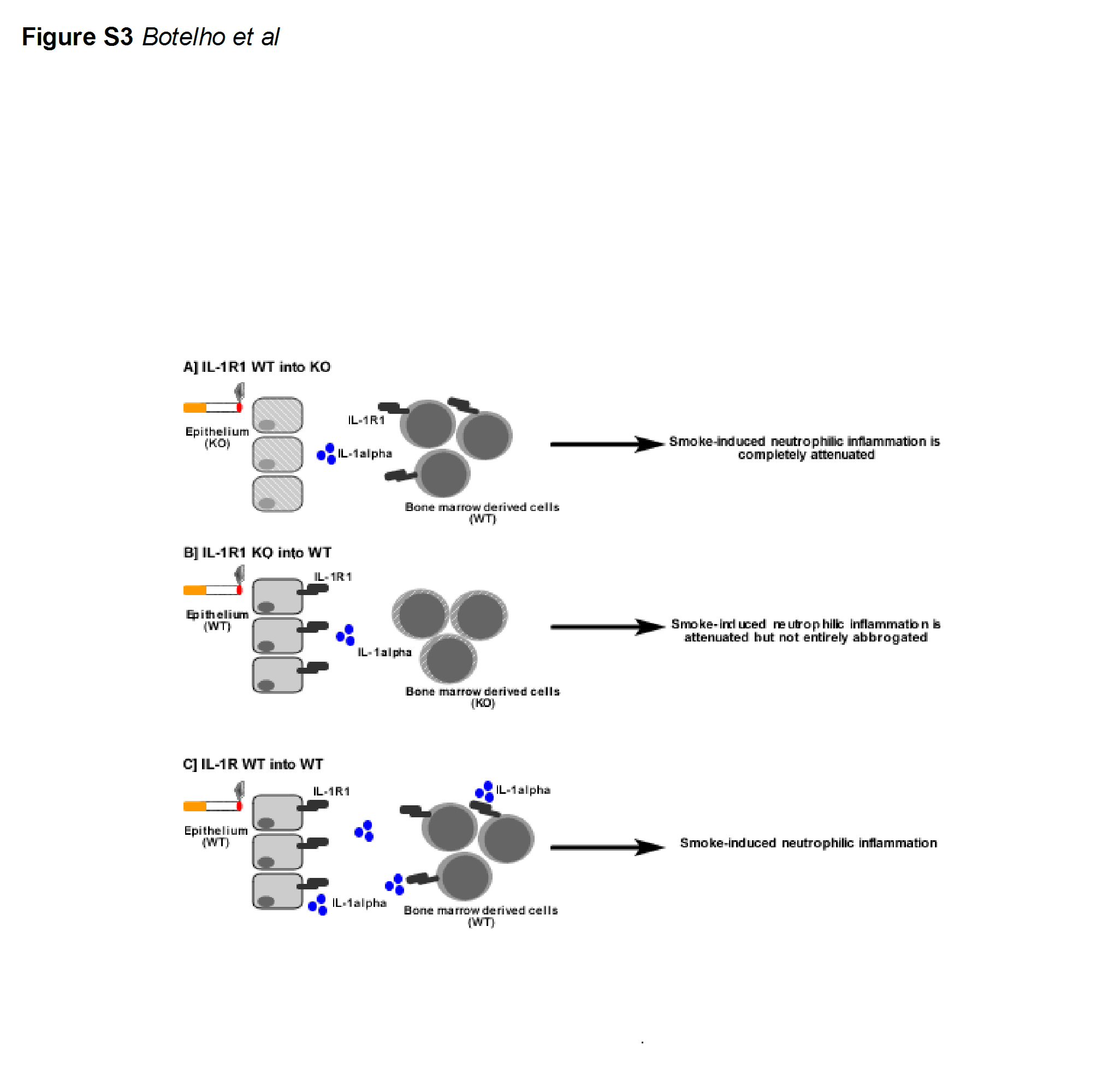

Supplement: Figure S3 — Model of IL-1α crosstalk between the hematopoietic and non-hematopoietic compartments. (A) Absence of the IL-1R1 on non-hematopoietic cells, as depicted on the airway epithelium completely attenuates smoke-induced inflammation. (B) Absence of the IL-1R1 on hematopoietic (macrophages and dendritic cells) leads to attenuated neutrophilic inflammation that is not entirely abrogated. (C) IL-1R1 is required on cells in both the hematopoietic and non- hematopoietic compartments for cross-talk to occur and maximal smoke-induced neutrophilic inflammation to result. (TIF) [file pone.0028457.s003.tif]

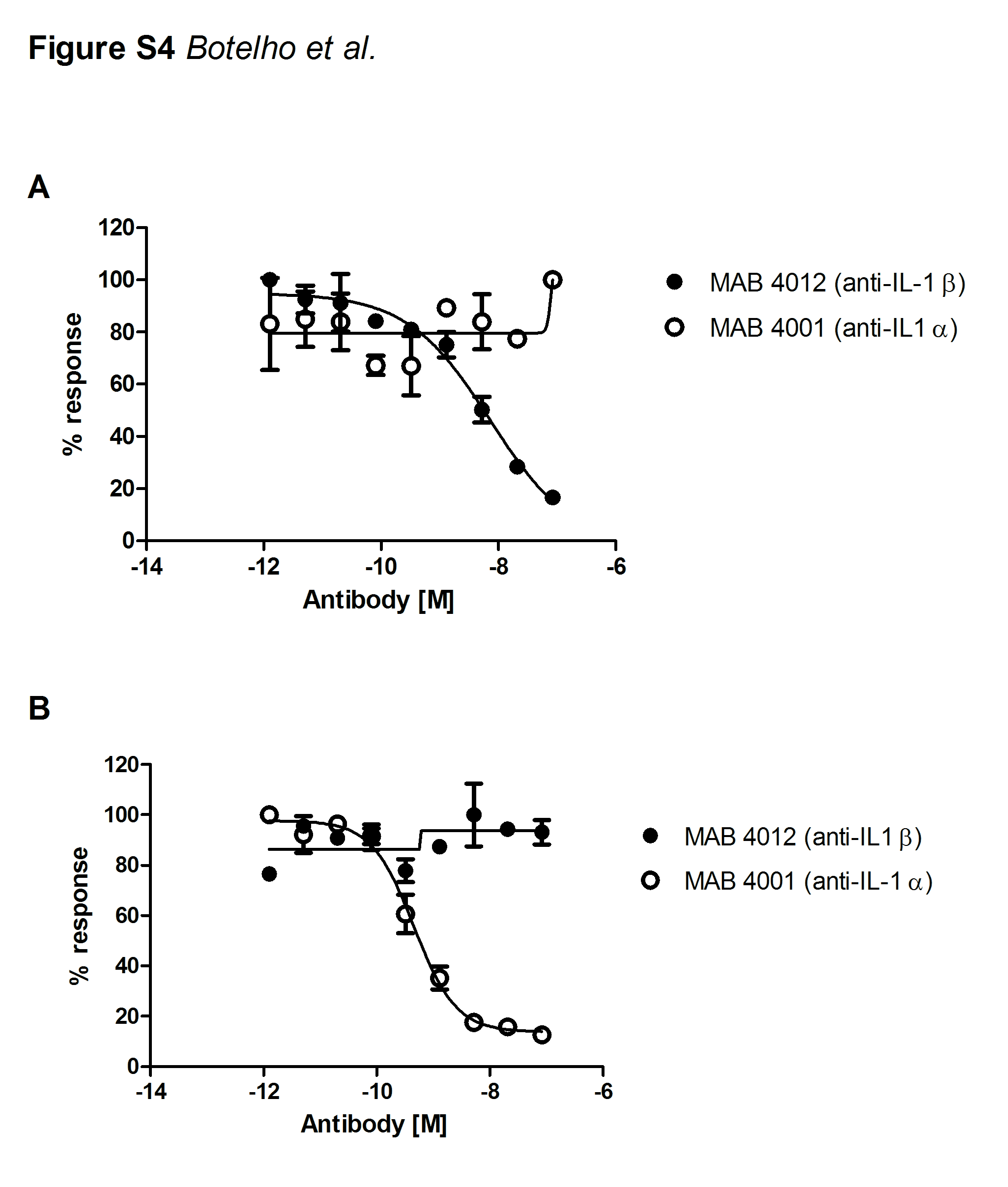

Supplement: Figure S4 — Antibody inhibition of IL-1 induced KC release from bEnd3 cells. Murine IL-1α and β induced KC release was measured from bEnd3 cells, a murine endothelial cell line. (A) Murine IL-1β (74 pM) induced KC release was inhibited by MAB4012, and not by MAB4001 (B) Murine IL-1α (74 pM) induced KC release was inhibited by MAB4001, but not by MAB4012. Data shown is a single experiment representative of n = 3. (TIF) [file pone.0028457.s004.tif]
