# Supplementary material for: IL-1α/IL-1R1 Expression in Chronic Obstructive Pulmonary Disease and Mechanistic Relevance to Smoke-Induced Neutrophilia in Mice
Source: PLoS One. 2011 Dec 6;6(12):e28457. doi: 10.1371/journal.pone.0028457 (PMC3232226; doi:10.1371/journal.pone.0028457)
Supplement: Table S1 — Study population characteristics, ( n = 14), for sputum cohort. (DOC) [file pone.0028457.s005.doc]

**Table S1. Study population characteristics, (*n* = 14), for sputum cohort**

| **Patient demographics at baseline visit** | | | | |
| --- | --- | --- | --- | --- |
|  | | **Substrata** | |  |
|  |  | |  | |
| **Gender** | | Male | | 11 |
|  | | Percent | | 78 |
|  | | Female | | 3 |
|  | | Percent | | 22 |
| **Current Smokers** | | Number | | 3 |
|  | | Percent | | 21 |
| **Pack years** | | Mean | | 49.5 |
|  | | (SD) | | (21.9) |
| **FEV_1_ %** | | Mean | | 35.8 |
|  | | (SD) | | (8.9) |
| **FEV_1_/FVC** | | Mean | | 40.1 |
|  | | (SD) | | (12.6) |
| **O_2_ Sat** | | Mean | | 93.7 |
|  | | (SD) | | (2.5) |
