# Supplementary material for: IL-1α/IL-1R1 Expression in Chronic Obstructive Pulmonary Disease and Mechanistic Relevance to Smoke-Induced Neutrophilia in Mice
Source: PLoS One. 2011 Dec 6;6(12):e28457. doi: 10.1371/journal.pone.0028457 (PMC3232226; doi:10.1371/journal.pone.0028457)
Supplement: Table S2 — Expression profile of various mediators in IL-1R1-deficient animals. (DOC) [file pone.0028457.s006.doc]

Table S2. Expression profile of various mediators in IL-1R1-deficient animals

| **Inflammatory mediator expression profiles** | | | | | | | | | | |  | |
| --- | --- | --- | --- | --- | --- | --- | --- | --- | --- | --- | --- | --- |
|  | **Substrata** | | | **Treatment Groups** | | | | | | | | |
|  | |  | | **Room air** | | |  | | **Smoke** | | | |
|  | |  | | **Wild-type** |  | **IL-1R1 KO** | |  | **Wild-type** |  | | **IL-1R1 KO** |
| Neutrophil-recruiting chemokines | |  | |  |  |  | |  |  |  | |  |
| CXCL-1 | | Mean | | 1.0 |  | 0.6 | |  | 7.9* |  | | 0.8† |
|  | | (SEM) | | (0.06) |  | (0.04) | |  | (0.4) |  | | (0.05) |
| CXCL-2 | | Mean | | 1.0 |  | 1.1 | |  | 12.8* |  | | 2.1† |
|  | | (SEM) | | (0.05) |  | (0.05) | |  | (0.4) |  | | (0.05) |
| CXCL-5 | | Mean | | 1.0 |  | 0.4 | |  | 21.2* |  | | 0.2† |
|  | | (SEM) | | (0.07) |  | (0.03) | |  | (0.8) |  | | (0.01) |
| Other  inflammatory  chemokines | | |  |  |  |  | |  |  |  | |  |
| CXCL-10 | | Mean | | 1.0 |  | 1.4 | |  | 2.1* |  | | 1.3† |
|  | | (SEM) | | (0.06) |  | (0.09) | |  | (0.09) |  | | (0.05) |
| CCL-2 | | Mean | | 1.0 |  | 1.4 | |  | 6.5* |  | | 1.6† |
|  | | (SEM) | | (0.05) |  | (0.06) | |  | (0.2) |  | | (0.03) |
| CCL-3 | | Mean | | 1.0 |  | 1.2 | |  | 4.3* |  | | 2.0† |
|  | | (SEM) | | (0.05) |  | (0.06) | |  | (0.03) |  | | (0.09) |
| CCL-4 | | Mean | | 1.0 |  | 1.1 | |  | 1.5 |  | | 1.0† |
|  | | (SEM) | | (0.07) |  | (0.05) | |  | (0.07) |  | | (0.01) |
| CCL-7 | | Mean | | 1.0 |  | 1.4 | |  | 5.8* |  | | 2.1† |
|  | | (SEM) | | (0.03) |  | (0.06) | |  | (0.4) |  | | (0.07) |
| CCL-9 | | Mean | | 1.0 |  | 0.9 | |  | 2.0* |  | | 0.8† |
|  | | (SEM) | | (0.04) |  | (0.01) | |  | (0.05) |  | | (0.02) |
| Inflammatory  cytokines | | |  |  |  |  | |  |  |  | |  |
| IL-1β | | Mean | | 1.0 |  | 0.8 | |  | 0.8 |  | | 0.6 |
|  | | (SEM) | | (0.07) |  | (0.01) | |  | (0.03) |  | | (0.02) |
| GM-CSF | | Mean | | 1.0 |  | 1.0 | |  | 3.6* |  | | 1.4† |
|  | | (SEM) | | (0.01) |  | (0.03) | |  | (0.06) |  | | (0.01) |
| TNFα | | Mean | | 1.0 |  | 1.1 | |  | 3.0* |  | | 1.1† |
|  | | (SEM) | | (0.01) |  | (0.03) | |  | (0.1) |  | | (0.03) |
| IL-6 | | Mean | | 1.0 |  | 1.0 | |  | 1.2 |  | | 0.6† |
|  | | (SEM) | | (0.05) |  | (0.06) | |  | (0.06) |  | | (0.02) |
| Proteinases | |  | |  |  |  | |  |  |  | |  |
| MMP-9 | | Mean | | 1.0 |  | 1.0 | |  | 0.9 |  | | 0.8 |
|  | | (SEM) | | (0.07) |  | (0.03) | |  | (0.05) |  | | (0.02) |
| MMP-12 | | Mean | | 1.0 |  | 0.9 | |  | 5.9* |  | | 1.2† |
|  | | (SEM) | | (0.04) |  | (0.02) | |  | (0.2) |  | | (0.02) |
| TIMP-1 | | Mean | | 1.0 |  | 1.1 | |  | 4.4* |  | | 1.5† |
|  | | (SEM) | | (0.05) |  | (0.05) | |  | (0.2) |  | | (0.05) |
| Receptors | |  | |  |  |  | |  |  |  | |  |
| CXCR1 | | Mean | | 1.1 |  | 1.5 | |  | 16.2* |  | | 25.7† |
|  | | (SEM) | | (0.07) |  | (0.06) | |  | (0.2) |  | | (0.3) |
| CXCR2 | | Mean | | 1.0 |  | 0.8 | |  | 1.4 |  | | 1.0† |
|  | | (SEM) | | (0.05) |  | (0.02) | |  | (0.06) |  | | (0.04) |
| Acute Phase Proteins | | |  |  |  |  | |  |  |  | |  |
| SAA-3 | | Mean | | 1.0 |  | 1.0 | |  | 154.6* |  | | 1.7† |
|  | | (SEM) | | (0.06) |  | (0.06) | |  | (12.1) |  | | (0.06) |
| Others | |  | |  |  |  | |  |  |  | |  |
| TGF-β1 | | Mean | | 1.0 |  | 0.9 | |  | 1.0 |  | | 1.0 |
|  | | (SEM) | | (0.01) |  | (0.01) | |  | (0.01) |  | | (0.01) |
| *p<0.05, comparison of wild-type, room air versus smoke  †p<0.05, comparison of smoke, wild-type versus IL-1R1 KO | | | | | | | | | | | | |
