# Supplementary material for: IL-1α/IL-1R1 Expression in Chronic Obstructive Pulmonary Disease and Mechanistic Relevance to Smoke-Induced Neutrophilia in Mice
Source: PLoS One. 2011 Dec 6;6(12):e28457. doi: 10.1371/journal.pone.0028457 (PMC3232226; doi:10.1371/journal.pone.0028457)
Supplement: Table S3 — Expression profile of various mediators following anti-IL-1 –α and –β intervention. (DOC) [file pone.0028457.s007.doc]

**Table S3. Expression profile of various mediators following anti-IL-1 –α and –β intervention.**

| **Inflammatory mediator expression profiles** | | | | | | | | | | |  | | |
| --- | --- | --- | --- | --- | --- | --- | --- | --- | --- | --- | --- | --- | --- |
|  | **Substrata** | | **Treatment Groups** | | | | | | | | | | |
|  |  | | **Room air** | |  | **Smoke** | | | | | | | |
|  |  | | **No Rx** |  | | **No Rx** |  | **IgG Isotype** |  | **Anti**  **IL-1α** | |  | **Anti**  **IL-1β** |
| Neutrophil-recruiting chemokines |  | |  |  | |  |  |  |  |  | |  |  |
| CXCL-1 | Mean | | 1.1 |  | | 8.9* |  | 7.3 |  | 2.1† | |  | 7.9 |
|  | (SEM) | | (0.09) |  | | (0.5) |  | (0.2) |  | (0.3) | |  | (0.1) |
| CXCL-2 | Mean | | 1.1 |  | | 7.3* |  | 5.8 |  | 3.1† | |  | 6.7 |
|  | (SEM) | | (0.1) |  | | (0.4) |  | (0.2) |  | (0.3) | |  | (0.2) |
| CXCL-5 | Mean | | 1.0 |  | | 8.4* |  | 7.1 |  | 1.1† | |  | 6.6 |
|  | (SEM) | | (0.06) |  | | (0.5) |  | (0.6) |  | (0.2) | |  | (0.6) |
| Other  inflammatory  chemokines | |  |  |  | |  |  |  |  |  | |  |  |
| CXCL-10 | Mean | | 1.0 |  | | 4.7* |  | 3.5 |  | 1.7† | |  | 4.3 |
|  | (SEM) | | (0.05) |  | | (0.3) |  | (0.2) |  | (0.09) | |  | (0.4) |
| CCL-2 | Mean | | 1.0 |  | | 24.4* |  | 18.8 |  | 4.6† | |  | 17.5 |
|  | (SEM) | | (0.04) |  | | (1.5) |  | (0.6) |  | (0.4) | |  | (0.6) |
| CCL-3 | Mean | | 1.0 |  | | 8.1* |  | 5.0 |  | 3.0† | |  | 6.1 |
|  | (SEM) | | (0.03) |  | | (0.3) |  | (0.1) |  | (0.2) | |  | (0.1) |
| CCL-4 | Mean | | 1.0 |  | | 4.5* |  | 3.5 |  | 1.0† | |  | 3.4 |
|  | (SEM) | | (0.01) |  | | (0.2) |  | (0.1) |  | (0.03) | |  | (0.09) |
| CCL-7 | Mean | | 1.1 |  | | 12.3* |  | 11.0 |  | 2.9† | |  | 10.0 |
|  | (SEM) | | (0.09) |  | | (0.8) |  | (0.5) |  | (0.2) | |  | (0.3) |
| CCL-9 | Mean | | 1.0 |  | | 4.7* |  | 5.0 |  | 2.5† | |  | 4.5 |
|  | (SEM) | | (0.03) |  | | (0.2) |  | (0.07) |  | (0.1) | |  | (0.1) |
| Inflammatory  cytokines | |  |  |  | |  |  |  |  |  | |  |  |
| IL-1β | Mean | | 1.0 |  | | 1.8* |  | 1.6 |  | 0.7† | |  | 1.6 |
|  | (SEM) | | (0.05) |  | | (0.06) |  | (0.05) |  | (0.04) | |  | (0.02) |
| GM-CSF | Mean | | 1.0 |  | | 3.4* |  | 2.9 |  | 1.7† | |  | 2.9 |
|  | (SEM) | | (0.02) |  | | (0.09) |  | (0.07) |  | (0.06) | |  | (0.06) |
| TNFα | Mean | | 1.0 |  | | 4.8* |  | 3.9 |  | 2.2† | |  | 3.4 |
|  | (SEM) | | (0.03) |  | | (0.2) |  | (0.2) |  | (0.08) | |  | (0.08) |
| IL-6 | Mean | | 1.0 |  | | 5.7* |  | 5.5 |  | 2.3† | |  | 5.8 |
|  | (SEM) | | (0.05) |  | | (0.4) |  | (0.4) |  | (0.2) | |  | (0.4) |
| Proteinases |  | |  |  | |  |  |  |  |  | |  |  |
| MMP-9 | Mean | | 1.0 |  | | 1.4* |  | 1.2 |  | 0.8† | |  | 1.3 |
|  | (SEM) | | (0.03) |  | | (0.04) |  | (0.05) |  | (0.04) | |  | (0.05) |
| MMP-12 | Mean | | 1.0 |  | | 7.9* |  | 7.7 |  | 3.3† | |  | 0.1 |
|  | (SEM) | | (0.02) |  | | (0.2) |  | (0.1) |  | (0.1) | |  | (0.1) |
| TIMP-1 | Mean | | 1.0 |  | | 6.8* |  | 6.3 |  | 1.8† | |  | 6.1 |
|  | (SEM) | | (0.03) |  | | (0.4) |  | (0.4) |  | (0.1) | |  | (0.2) |
| Receptors |  | |  |  | |  |  |  |  |  | |  |  |
| CXCR1 | Mean | | 1.0 |  | | 2.6* |  | 2.4 |  | 4.0† | |  | 2.5 |
|  | (SEM) | | (0.05) |  | | (0.07) |  | (0.1) |  | (0.07) | |  | (0.08) |
| CXCR2 | Mean | | 1.0 |  | | 1.7* |  | 1.5 |  | 0.8† | |  | 1.5 |
|  | (SEM) | | (0.04) |  | | (0.03) |  | (0.04) |  | (0.03) | |  | (0.03) |
| Acute Phase Proteins | |  |  |  | |  |  |  |  |  | |  |  |
| SAA-3 | Mean | | 1.1 |  | | 335* |  | 320 |  | 12.1† | |  | 288 |
|  | (SEM) | | (0.09) |  | | (25.6) |  | (21.4) |  | (1.4) | |  | (16.2) |
| Others |  | |  |  | |  |  |  |  |  | |  |  |
| TGF-β1 | Mean | | 1.0 |  | | 1.1 |  | 1.1 |  | 1.0 | |  | 1.0 |
|  |  | | (0.01) |  | | (0.02) |  | (0.01) |  | (0.02) | |  | (0.02) |
| *p<0.05, comparison of smoke, no rx to room air, no rx  †p<0.05, comparison of smoke, anti-IL-1α to smoke, no rx | | | | | | | | | | | | | |
